# Image Analysis of Sewage Sludge and Barley Straw as Biological Materials Composted under Different Conditions

**DOI:** 10.3390/ma12223644

**Published:** 2019-11-06

**Authors:** Sebastian Kujawa, Damian Janczak, Andrzej Mazur

**Affiliations:** 1 Institute of Biosystems Engineering, Poznań University of Life Sciences, Wojska Polskiego 50, Poznań 60–627, Poland; damian.janczak@up.poznan.pl; 2 Department of Environmental Engineering and Geodesy, University of Life Sciences in Lublin, Leszczyńskiego 7, Lublin 20–069, Poland; amazur70@op.pl

**Keywords:** image analysis, composting, bioreactors, sewage sludge, barley straw

## Abstract

Composting is one of the most important methods of sewage sludge management. This paper describes the methods of computer image analysis used for objective comparison of the appearance of composted materials under diverse conditions in terms of size and thermal insulation of the composting chambers. The research material was a mixture of sewage sludge and barley straw. The composting process was performed under strictly controlled laboratory conditions, using 10 composting chambers with five different volumes. In half of them additional thermal insulation was used, while in the others no insulation was applied. A proper composting process run was observed only in the three chambers with the largest volume and with additional thermal insulation. The images of the materials were subjected to a wide analysis, wherein the values of 17 parameters regarding color and texture were estimated. Significant differences were observed in the appearances between materials obtained during the properly running composting processes and those obtained in the chambers of insufficient size and thermal insulation. The values of the considered parameters determined for images of the composted material under normal and abnormal conditions were significantly different from each other. Thus, these parameters may be used as indicators of a correctly conducted composting process. In the cases of 15 parameters, the values of these differences exceeded 10%, and in the cases of 10 parameters 50%, while in the cases of three parameters as much as 100%.

## 1. Introduction

Composting is the process of organic matter decomposition by aerobic microorganisms, commonly used both in home applications and on an industrial scale. During this process, under the influence of microorganisms, composted biomass is significantly heated [1]. This situation is conducive to the destruction of morbific pathogens contained in its interior [2], and under appropriate conditions may lead to its pasteurization [3,4]. Therefore, materials that due to unfavorable microbiological composition (in the unprocessed form) are often harmful to humans and other living organisms are often subjected to the composting process. An example is municipal sewage sludge [5,6,7,8]. Unfortunately, due to the amorphous structure and unfavorable chemical composition (excess of nitrogen in relation to carbon), the use of sewage sludge as the only substrate for composting is not rational [9,10]. Therefore, it is mixed with other substrates that loosen the structure and at the same time improve the carbon (C) to nitrogen (N) ratio [11,12]. Such material may be, for example, cereal straw, including barley, rape straw, sawdust as waste from wood processing, grass or leaves. Composting of the material containing sewage sludge in the right way, on the one hand, it promotes the hygienization of this sludge depriving it of unfavorable features, often manifesting as unpleasant odors, and on the other hand, it allows valuable fertilizer material to be obtained [13,14].

In Poland, composting of sewage sludge is particularly important in the light of legal regulations and standards introduced by the European Union. These regulations prohibit the basic method of managing sludge, that is, storage. As a result, composting, next to the combustion process, will become one of the two most important methods of this type of waste management. This fact has made it possible to observe recently increased research activity in the field of sewage sludge composting [9,15,16,17,18,19,20], related to both recognizing these processes and their optimization in terms of minimizing the time needed to obtain a final product with adequate quality. The research on composting of municipal sewage sludge is most often carried out on a laboratory scale. Composting chambers, also called bioreactors, are used for this purpose, which allow monitoring and controlling of the processes [11,21,22,23]. During such tests a number of physicochemical parameters are monitored and analyzed, including the following [11,21]:

- batch temperature,
- concentration of oxygen and carbon dioxide in the outgoing air,
- emissions of ammonia, hydrogen sulfide and methane,
- content of mineral and organic matter, mass of dry matter, pH, and conductivity of the material.

Knowledge of these parameters allows us to conclude on the correctness of the composting process, as well as on the sufficient maturity of the material and the possibility of completing the process.

In a properly conducted process, a thermophilic phase appears in which the material temperature exceeds 45 °C and can reach even 80 °C. Furthermore, it is also possible to observe a clear decrease in the oxygen concentration in the air coming out of the bioreactor chambers, which is a result of the increased activity of aerobic bacteria that generate CO_2_ as a product of the decomposition of organic matter. At the end of the process, the material temperature drops below 30 °C, and the oxygen concentration in the outgoing air approaches the nominal level. The distribution of organic matter occurring during composting causes a clear change in the structure and appearance of the material. On the basis of the changes in the compost appearance, it is possible to assess its maturity degree [17]. An objective assessment of these changes, however, requires the use of computer image analysis methods [24,25,26]. Such methods have turned out to give an adequate and reliable tool supporting the classification and assessment of the condition and composition of various materials. They are used in the field of waste management [27,28,29] as well as in broadly defined agriculture [30,31,32,33] and horticulture [34,35].

Running the composting process of the same material under different conditions may cause the properties of the product obtained after a certain time to be different. As a result, the appearance of this product may be different, and sometimes even far from the appearance required for the commercialization of the compost (brown, flowing, and homogeneous material). It can be assumed that the use of computer image analysis methods, in particular, color and texture analysis, will allow an objective assessment of these differences. Therefore, the purposes of this work were as follows:

- to use computer image analysis methods to objectively compare the appearance of material composted under different conditions in terms of the size and thermal insulation of the composting chambers,
- to determine whether the image parameters of the composted material may be useful as an indicator of composting correctness.

## 2. Materials and Methods 

### 2.1. Research Material

The research material subjected to composting was a mixture of sewage sludge and barley straw in proportions of 65% to 35% in relation to the content of dry substance. The substrates were produced in the Wielkopolska Voivodship, near Poznań (Poland). Sewage sludge came from the municipal sewage treatment plant in Szamotuły (a city of less than 40,000 inhabitants, without heavy industry) while the straw came from the branch of the Agricultural Experimental Farm Swadzim, located in Złotniki, belonging to the Poznań University of Life Sciences.

The composting process was performed under strictly controlled laboratory conditions. The experiments were carried out in 10 composting chambers with 5 different volumes, i.e. 10, 20, 50, 74, and 119 dm^3^. Two variants of the chambers were used for each volume, with the use of additional thermal insulation and without. This insulation was made of mineral wool with a thickness of 150 mm and a thermal conductivity coefficient of 0.04 W ∙ (m ∙ K)^−1^. Application of additional insulation was aimed at better mapping of the conditions prevailing inside the compost pile in industrial composting plants.

The duration of the process was 22 days. The diagram of the performed experiments is presented in Table 1. During the process, the following parameters were monitored: material temperature, concentration of oxygen, and carbon dioxide in the outgoing air as well as emissions of ammonia, hydrogen sulfide, and methane. After the completion of the processes, samples of material for image acquisition were taken as well as standard physicochemical analyses being carried out. These analyses included determination of such parameters as: dry substance content, pH, conductivity, organic matter content, ammonium, and general nitrogen as well as organic carbon [11,21]. To ensure a more representative character of the material samples, we assumed that they would be taken from the chambers in a random manner, after mixing the material.

The studies assumed that the appropriate quality material obtained in the composting process was compost at the early maturity stage. In reference to previous studies [17], the following criteria for achieving compost at this stage were taken into account:

- The obtained material should have a dark color and smell similar to the smell of horticultural soil or forest mulch, unacceptable is a smell of rot or a specific and unpleasant smell, resulting from the increased emission of ammonia or hydrogen sulfide.
- The tested material should undergo the hygienisation process, i.e., its temperature during the process should be at least 55 °C for at least 1 day or reach 70 °C for at least 1 h.
- The temperature of the material obtained at the end of the process should not be higher than 30 °C.
- The material should be relatively stable, in the air leaving the bioreactor chambers the oxygen content should be higher than 18%, and the content of carbon dioxide should not exceed 2.9%.
- The material pH of the at the end of the process should be between 7 and 9.

### 2.2. Image Acquisition

The basic element of the stand for image acquisition of composted material samples was a photographic chamber illuminated with visible light [36]. The sample images recorded in visible light corresponded to what would be seen by the human eye in the form of light reflected from the material under standard lighting conditions, e.g., in sunlight. The use of the photographic chamber allowed the image acquisition of the analyzed material to be performed always under the same lighting conditions. This was necessary considering the need to obtain objective values of the parameter from the images.

**Figure 1:**
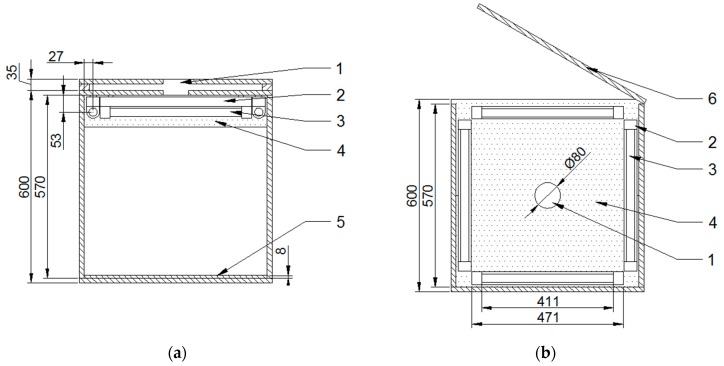
Diagram of the photographic chamber construction (dimensions in millimeters): (**a**) chamber interior view from the side, (**b**) view of the chamber upper inner part, 1—lens hole, 2—fluorescent lamp fixture, 3—fluorescent lamp, 4—reflector film, 5—extendable tray for material, 6—door [36].

The internal dimensions of the photographic chamber were 570 mm × 570 mm × 570 mm. The photographed material was placed on a pull-out tray located at the bottom of the chamber. The upper wall of the chamber was equipped with a hole that allowed the lens of the camera to be placed. The light source was 4 fluorescent lamps Sylvania Luxline Plus F15W/865 (Feilo Sylvania, Budapest, Hungary) embedded in fluorescent lamp fixtures placed in the upper part of the chamber. These lamps emit visible light with a color temperature of 6500K (cold daylight) and have a high color rendering index Ra of 85 (class 1B). The upper inner surface of the chamber and its internal side surfaces up to a height of 100 mm from the top, as well as the fixtures, were covered with a reflector foil increasing the amount of light reflected in the direction of the photographed material. Other interior surfaces were painted black to minimize the light reflection in undesirable directions. The diagram of the photographic chamber construction is presented in Figure 1.

An important element of the image acquisition station was the acquisition equipment. It included the following elements:

- DSLR (digital single-lens reflex) camera with DX format and 10 megapixel resolution: Nikon D80 (Nikon Corporation, Tokyo, Japan),
- 35 mm focal length lens (DX format): Nikkor 35 mm f/1.8G AF-S DX (Nikon Corporation, Tokyo, Japan),
- high efficiency UV filter: Hoya Super HMC Pro1 (HOYA Corporation, Tokyo, Japan),
- Silk Goodman Digital tripod (SLIK Corporation, Hidaka City, Japan), allowing for lower suspension of the camera.

In order to minimize the possibility of noise in the image, the camera ISO sensitivity was set to the lowest of the available values, i.e., ISO 100 [37]. The aperture value was set to f/5.6, and according to the photographic principles, the exposure time was set to 1/25 s. Both the exposure time as well as the correct white balance value were determined manually according to the amount and color temperature of visible light illuminating the photographed material. For this purpose, a light meter built into the body of the camera and a model gray photo card Lastolite EzyBalance LR 1250 (Manfrotto, Cassola, Italy), reflecting 18% of visible light was used. Photographs were taken in raw format (NEF) and then converted into JPG files.

### 2.3. Image Processing and Analysis

From the material produced as part of each of the 10 composting processes carried out, 32 images with a resolution of 968 × 648 pixels corresponding to an area of approx. 98 mm × 65 mm were obtained. For each image, 17 values for color and texture were determined. In order to determine a part of them, the following transformations were necessary [17]:

- converting an image from a 24-bit RGB model to an 8-bit grayscale using the weighted sum of the component values R, G, and B:
  (1) Brightness=0.2989·R+0.5870·G+0.1140·B.
- binarization of the image taking into account 4 threshold values, i.e. 0.05, 0.10, 0.15, and 0.20 (in the range from 0 to 1).

GLCMs (grey level co-occurence matrices) were determined in the texture analysis process [17,38,39,40,41,42]. The following parameters were adopted when determining these matrices:

- eight brightness classes of a pixel,
- neighborhood in the form of 1 pixel,
- four directions of neighborhood analysis: 0°, 45°, 90°, and 135° (symmetrically).

Finally, the values of the following color parameters were determined:

- R_MEAN, G_MEAN, and B_MEAN - the average brightness value of the R, G, and B components of a pixel in a 24-bit image in the RGB model,
- R_MEDIAN, G_MEDIAN, and B_MEDIAN—median brightness of R, G, and B components of a 24-bit pixel on an image in the RGB model,
- GS_MEAN—the average brightness value of a pixel on an 8-bit grayscale image,
- GS_MEDIAN—median brightness of a pixel on an 8-bit grayscale image,
- WH_PERCENT1, WH_PERCENT2, WH_PERCENT3, and WH_PERCENT4—percentage share of white color in the binarized image, respectively for the binarization thresholds 0.05, 0.10, 0.15, and 0.20.

In turn, in relation to the texture, the values of the following parameters were defined:

- ENTROPY—entropy of an 8-bit grayscale image,
- CONTRAST—brightness contrast between pixels and their neighborhood on an 8-bit grayscale image, averaged for selected directions,
- CORRELATION—correlation between pixels and their neighborhood on an 8-bit grayscale image, averaged for selected directions,
- ENERGY—energy for an 8-bit grayscale image, averaged for selected directions,
- HOMOGENEITY—uniformity of an 8-bit grayscale image, averaged for selected directions.

To determine the values of texture parameters based on the designated GLCMs, the following formulas were used [17,38,39,40,43]:

(2) $$Entropy=-\overset{256}{\underset{k=1}{\sum }}\left(n_{k}\cdot log_{2}n_{k}\right),\;$$

(3) $$Contrast=\overset{8}{\underset{i=1}{\sum }}\overset{8}{\underset{j=1}{\sum }}{\left(i-j\right)}^{2}p\left(i,j\right),$$

(4) $$Correlation=\overset{8}{\underset{i=1}{\sum }}\overset{8}{\underset{j=1}{\sum }}\frac{\left(i-\mu_{i}\right)\left(j-\mu_{j}\right)p\left(i,j\right)}{\sigma_{i}\sigma_{j}},$$

(5) $$Energy=\overset{8}{\underset{i=1}{\sum }}\overset{8}{\underset{j=1}{\sum }}p{\left(i,j\right)}^{2},$$

(6) $$Homogeneity=\overset{8}{\underset{i=1}{\sum }}\overset{8}{\underset{j=1}{\sum }}\frac{p\left(i,j\right)}{1+\left(i-j\right)},$$

where:

(7) $$\mu_{i}=\overset{8}{\underset{i=1}{\sum }}\overset{8}{\underset{j=1}{\sum }}i\cdot p\left(i,j\right),$$

(8) $$\mu_{j}=\overset{8}{\underset{i=1}{\sum }}\overset{8}{\underset{j=1}{\sum }}j\cdot p\left(i,j\right),$$

(9) $$\sigma_{i}=\sqrt{\overset{8}{\underset{i=1}{\sum }}\overset{8}{\underset{j=1}{\sum }}{\left(i-\mu_{i}\right)}^{2}\cdot p\left(i,j\right)},$$

(10) $$\sigma_{i}=\sqrt{\overset{8}{\underset{i=1}{\sum }}\overset{8}{\underset{j=1}{\sum }}{\left(j-\mu_{j}\right)}^{2}\cdot p\left(i,j\right)},$$

*n_k—_*number of pixels with k brightness, *i—*row number of the GLCM, *j—*column number of the GLCM, *p(i, j)—*value of the GLCM element with indices *(i, j)* divided by the sum of all elements.

The texture parameters, i.e. entropy, contrast, energy and homogeneity, were firstly determined individually for the four GLCMs obtained for selected directions of neighborhood. Then the values of these parameters were averaged.

Acquisition of parameters regarding the color and texture contained in images of composted material samples was carried out in an automated manner using the proprietary Compost Image Analysis software (version 1.5, Sebastian Kujawa, Poznań, Poland), [17]. It was created in the MATLAB environment extended with the Image Processing Toolbox.

## 3. Results and Discussion

### 3.1. The Course of the Composting Process and Analysis of the Obtained Material

Table 2 presents selected physicochemical parameters of the material obtained at the end of the composting experiments. After their analysis, and especially after assessing the characteristics of temperature changes and oxygen concentration, it was apparent that these processes were very diverse. Taking into account the assumptions made, it was found that only three out of the 10 experiments were carried out correctly. These were experiments marked with the symbols C50I, C74I and C119I, realized in chambers 50, 74, and 119 dm^3^ additionally insulated with mineral wool. Only in these experiments was the material temperature reached and maintained for at least 1 day at a level of at least 55 °C, which indicates a successful passage of the hygienization process. At the end of the composting process, the material temperature stabilized at a level below 30 °C. It is worth noting that in the initial phase of these three experiments a clear reduction in the oxygen concentration in the air coming out of the composting chambers was observed with the lowest value from about 11% to about 13% on the 5^th^ to 7^th^ day of the process. This low O_2_ concentration, together with the highest levels of temperature measured during the experiment, illustrate very intensive decomposition of organic matter in the most dynamic period of the thermophilic phase in the composting process. Strongly decreasing oxygen concentration is related to high microbiological activities during organic matter decomposition which lead to intensive production of CO_2_, H_2_O and heat.

**Figure 2:**
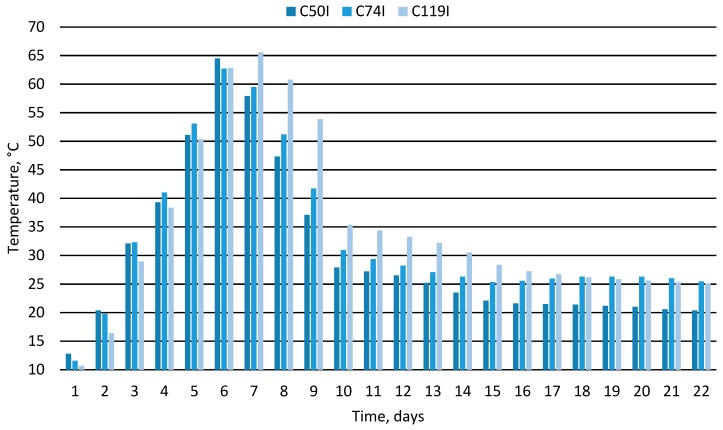
Change of composted material temperature over time.

**Figure 3:**
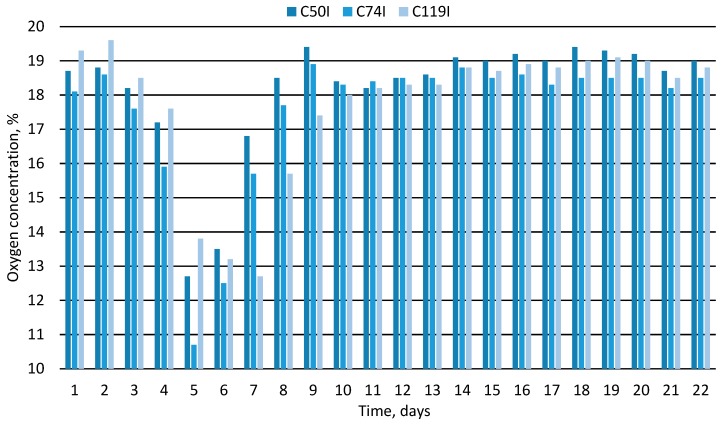
Change in oxygen concentration in the air coming out of composting chambers over time.

At the end of the process, only for these three experiments, the oxygen concentration in the outgoing air stabilized at a level higher than 18%, which in turn indicates the limited activity of aerobic bacteria and a significant inhibition of the oxygen decomposition of the material. Figure 2 and Figure 3 show the characteristics of temperature changes and oxygen concentration in the air coming out of the composting compartments for the three composting processes that went well. In the other seven composting variants, i.e., all of them carried out using chambers without additional thermal insulation (C10N, C20N, C50N, C74N, and C119N) and two realized using the smallest chambers in which additional insulation was applied (C10I and C20I), the composting process did not run properly. In these experiments, the composted biomass was not adequately heated and the oxygen content in the air did not stabilize at the appropriate level in the final phase of the process. It can therefore be concluded that the size of the composting chamber, as well as the method of its insulation leading (or not) to achieving the intense thermophilic phase, was important in terms of the correctness of the composting process.

**Figure 4:**
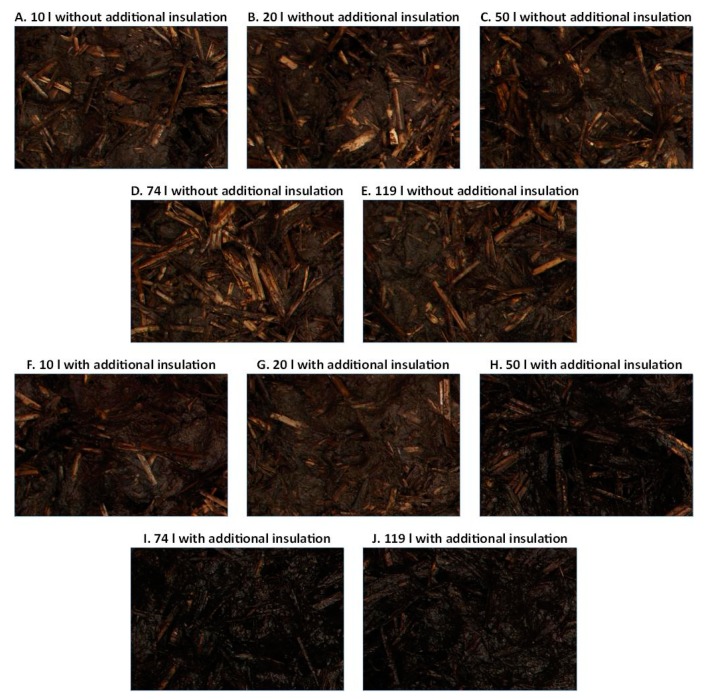
Exemplary images of samples of material produced in the considered composting chambers.

Figure 4 presents exemplary images of the composted material samples produced during the experiments. Even without using objective methods of computer image analysis, it can be seen that the images of the material produced in the C50I, C74I, and C119I chambers are darker than the images of the material obtained in the remaining chambers. This paper attempted an objective analysis of selected parameters describing the color and texture for images of material obtained in individual composting variants. The basis for this analysis were the values of the adopted parameters averaged for 32 images acquired for each of the 10 composting variants.

### 3.2. Color and Texture Analysis

The objective color analysis of the obtained images was first associated with the determination of parameters such as mean and median brightness of the pixel. These parameters were determined separately for individual components of images in the RGB model, as well as for images converted to the grayscale. Moreover, as part of the color analysis, grayscale images were binarized [44] using the four adopted threshold values, and then the percentage of white content on the binarized images was determined [17,40]. In the first place, the parameters indicated were determined for all the material images acquired as part of the subsequent composting experiments, and then the average values of these parameters for individual experiments were determined.

**Figure 5:**
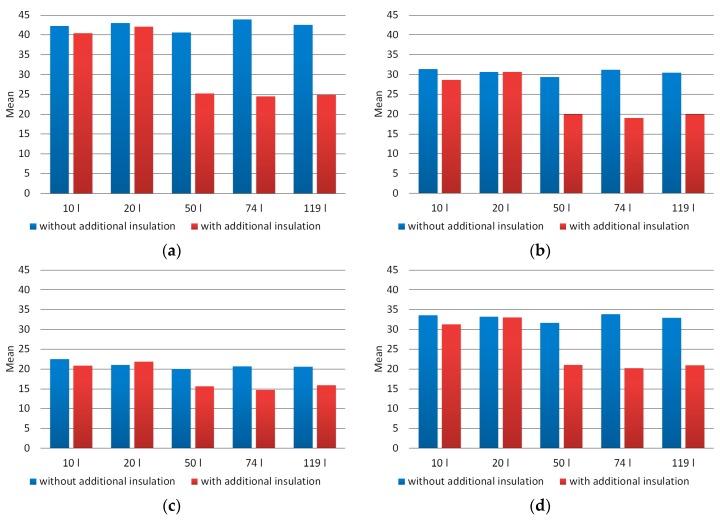
Mean brightness of the pixel averaged for images of composted material (**a**)—component R, (**b**)—component G, (**c**)—component B, (**d**)—grayscale.

**Figure 6:**
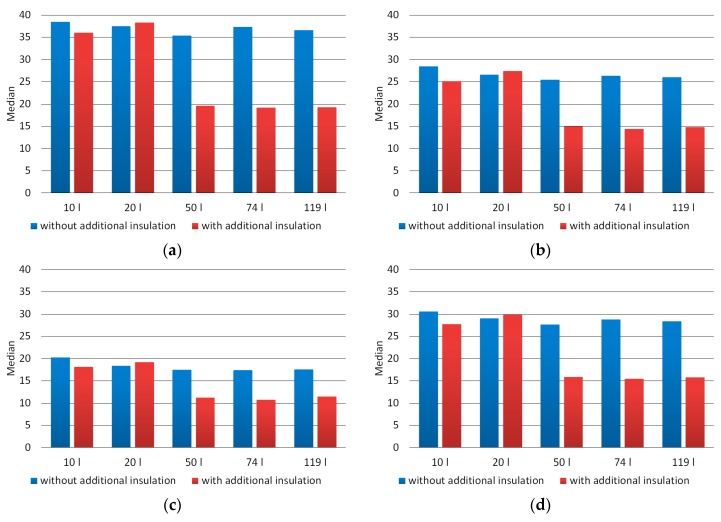
Median brightness of the pixel averaged for images of composted material (**a**)—component R, (**b**)—component G, (**c**)—component B, (**d**)—grayscale.

Figure 5 presents the charts of the mean brightness of the individual components of the pixel (R, G, and B), as well as the mean brightness of the pixel in the grayscale for images of the material produced in the composting chambers used. In contrast, Figure 6 presents the median brightness graphs of the pixel, including both image components in the RGB model and the grayscale. The averaged values of mean brightness of the R, G, B components for properly composted material (chambers 50, 74, and 119 dm^3^ with additional thermal insulation) were respectively 25, 20, and 15. In contrast, for incorrectly composted material they were successively 42, 30, and 21, so they were higher by respectively 69%, 55%, and 37%. The averaged median brightness values of the R, G, B components for properly composted material were successively 19, 15, and 11, while for incorrectly composted material amounted respectively 37, 27, and 18 and were higher by respectively 92%, 80%, and 65%. On analyzing the values of the mean and median brightness of the pixel for grayscale images, it was found that for properly composted material the values of these parameters were 21 and 16, respectively. In turn, for improperly composted material they were respectively: 33 and 29, and were therefore higher by respectively: 58% and 84%.

**Figure 7:**
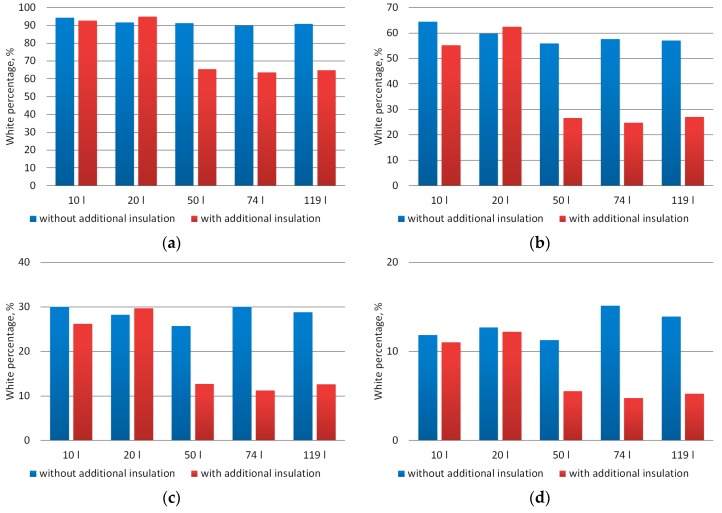
Percentage of white color averaged for images of composted material subjected to binarization (**a**)—threshold 0.05, (**b**)—threshold 0.10, (**c**)—threshold 0.15, (**d**)—threshold 0.20.

Figure 7 presents graphs of the percentage of white content on the binarized images of material produced in the considered composting chambers. For each of the assumed binarization thresholds one can see a significant difference in the whiteness content between the binary images of the material produced both under conducive and unfavorable conditions for composting. The averaged percentage of white color for images of correctly composted material was: 65%, 26%, 12%, and 5%, respectively for the binarization threshold: 0.05, 0.10, 0.15, and 0.20. In turn, for material composted under unfavorable conditions, the values of this parameter for subsequent binarization thresholds were 92%, 59%, 28%, and 13%, respectively, so they were relatively higher by 43%, 125%, 134%, and 143%, respectively.

Regarding the color analysis of the obtained material, carried out for the RGB model, grayscale and binarized images, there were clear differences between the values of each of the considered parameters specified for images of material obtained during correctly running composting processes (chambers with a volume of at least 50 dm^3^ with additional thermal insulation), and those defined for images of material for which insufficient size and thermal insulation of the composting chamber did not allow the proper course of the process. Regarding the images in the RGB model, it was found that the largest differences between the composted material under favorable conditions and those produced under unfavorable conditions occurred in relation to the red component (R), followed by the green (G) component and the blue one (B).

Analysis of the texture of the obtained images was associated with the determination of such parameters as: entropy, contrast, correlation, energy, and homogeneity. These parameters were determined for images converted to the grayscale. The values of the last four parameters were determined based on the GLCMs. First, the texture parameters were determined for all material images acquired as part of subsequent composting experiments, and then the average values of these parameters for individual experiments were determined.

**Figure 8:**
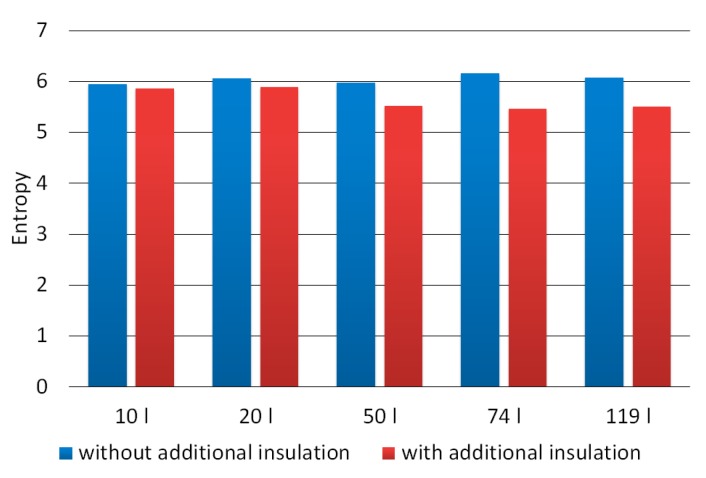
Entropy averaged for images of composted material in the grayscale.

Figure 8 shows the entropy chart for grayscale images of the material produced in the composting chambers used. The averaged value of entropy for properly composted material was 5.5. In turn, for incorrectly composted material, the value of this parameter amounted to 6.0 and was higher by 9%.

**Figure 9:**
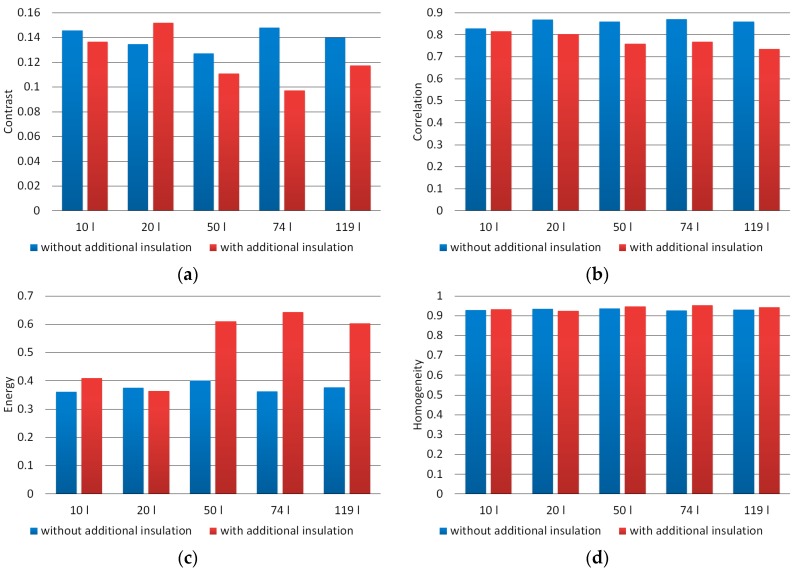
Texture parameters determined using an GLCMs for images of composted material in grayscale (**a**)—contrast, (**b**)—correlation, (**c**)—energy, (**d**)—homogeneity.

Figure 9 presents the values of texture parameters determined using the GLCMs. The average contrast value for composted material under favorable conditions was 0.11, while for composted material under unfavorable conditions it amounted to 0.14 and was 30% higher. In turn, the average correlation value for correctly composted material was 0.75, and for incorrectly composted material 0.84 and was higher by 12%. The biggest difference in texture parameters was observed in the energy range. The average value of this parameter for compost obtained under favorable conditions was 0.62, and for material obtained under unfavorable conditions it amounted to 0.38 and was lower by 39%. Regarding the homogeneity, it was noted that the differences between correctly and incorrectly composted materials were negligible. With respect to correctly composted material, the average value of this parameter was 0.95, while for incorrectly composted material it amounted 0.93 and was lower by 2%.

As a result of texture analysis performed for images of the obtained material, the differences between the values of the considered parameters specified for images of material obtained during properly running composting processes were observed, i.e. in chambers with size of at least 50 dm^3^ with additional thermal insulation and specified for material images, whose insufficient size and thermal insulation of the chamber did not allow the proper course of the process. Considering the highest values of these differences expressed as a percentage, the order of the texture parameters is as follows: energy, contrast, correlation, entropy, and homogeneity.

**Figure 10:**
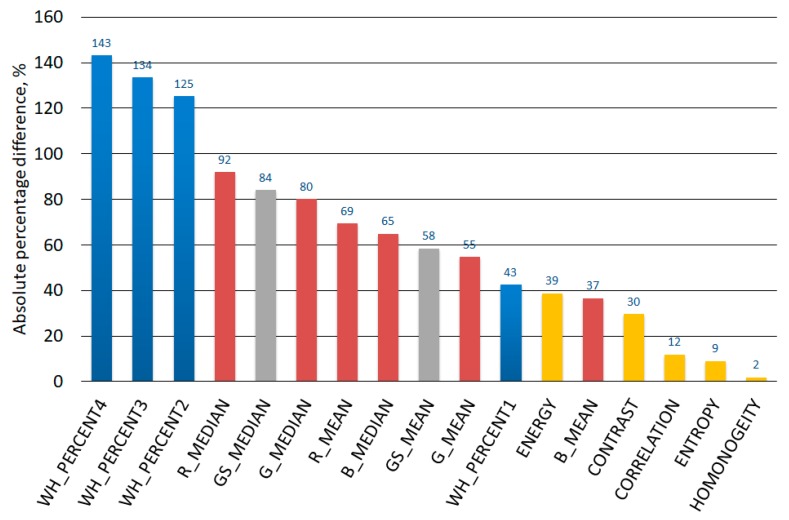
Absolute percentage differences in the values of the analyzed parameters of color and texture for the images of the material obtained under favorable and unfavorable composting conditions (blue color—parameters for a binarized image, red color—parameters for an RGB model image, gray color—parameters for a grayscale image, yellow color—texture parameters for a grayscale image).

Figure 10 presents the percentage of absolute differences between the values of 17 color and texture parameters specified for the composted material under favorable and unfavorable conditions. The graph shows that the highest values of these differences were characterized by three parameters concerning the percentage of white color in the images subjected to binarization, respectively for the binarization thresholds of 0.20, 0.15, and 0.10. Only for these parameters did the values of the analyzed differences significantly exceed 100%. With regard to the next seven parameters, the analyzed differences were higher than 50%. In order they were as follows: the median brightness of the R component, the median brightness of the image in the grayscale, the median brightness of the G component, the median brightness of the R component, the median brightness of the B component, the average brightness of the image in the grayscale, the average brightness of the G component. It is worth noting that these parameters concerned only the color in the RGB and grayscale models. The smallest percentage differences were recorded for four out of five texture parameters, namely: contrast, correlation, entropy, and homogeneity. Regarding the parameters of the texture, the greatest difference was shown by energy, which was in 12th place.

Statistical analyses were carried out for image parameters determined for material composted in favorable and unfavorable conditions. Average values of the parameters were estimated, as well as standard deviations. In addition, the existence of significant differences between the parameters was examined. For this purpose, the Mann–Whitney U test was used and the level of significance α = 0.05 was adopted. These differences were found to be highly significant for all of the parameters (*p*-values were very close to zero and definitely lower than α). The main results of the statistical analyses are given in Table 3.

In the properly running composting processes, the material temperature reached the highest values of up to about 65 °C, and for a period of a few days it remained at a definitely higher level than in the other chambers. Therefore, it can be assumed that ensuring the right temperature of the material is the key factor having the greatest impact on the production of good quality compost. The material obtained in compost chambers, in which the composting process proceeded correctly differed in appearance from the material obtained under unfavorable conditions. The differences in appearance were expressed in an objective manner using selected parameters of color and textures obtained from the images of samples of the obtained material. The differences of the analyzed parameters were statistically significant and they may be useful as an indicator of correctly conducted sewage sludge composting.

The studies carried out in this paper were pilot studies of a unique nature. The authors have not found any other studies in the literature aimed at an objective comparison of differences in the appearance of the material composted under different conditions in terms of the size and thermal insulation of the composting chambers. There are a few studies in which the methods of computer image analysis combined with neural modelling have been used to assess certain compost features, such as maturity degree [17,45] or dry matter content [46]. However, their objectives and methods are very far from the studies presented in this manuscript. Proper evaluation of the material obtained in the composting process requires knowledge of at least its physicochemical parameters, preferably in combination with the characteristics of changes in the material temperature and the chemical composition of gases leaving the compost chambers during the process [11,17]. Unfortunately, such analyses are expensive, time-consuming and in practice difficult to perform under real-life conditions of a composting plant. As this study has shown, methods based on computer image analysis may in the future provide an alternative, simplified approach to the approximate evaluation of the material obtained. However, for this to happen, further studies should be carried out. They should include more substrates to be analyzed, as well as more repetitions of composting experiments.

## 4. Conclusions

Correct composting conditions were achieved in three out of 10 chambers, i.e., those with a volume of 50, 74, and 119 dm^3^ with additional thermal insulation in the form of 150 mm thick mineral wool. The composting process did not run properly in the chambers where no additional thermal insulation was applied, and in the two smallest chambers (10 and 20 dm^3^), where such insulation was used. The values of 17 considered parameters determined for images of composted material under correct and abnormal conditions were significantly different. They may be used as indicators of correctly conducted composting process. In the case of 15 parameters, the value of these differences exceeded 10%. The biggest differences, definitely exceeding 100%, were recorded for three parameters regarding the percentage of white color in the image subjected to binarization with the threshold values: 0.20, 0.15, and 0.10. Statistical parameters determined for the brightness of individual components of the image in the RGB model and the brightness of the grayscale image were characterized by differences in the range of 37% to 92%. The differences considered for the analyzed texture parameters belonged to the least clear ones. While these differences in terms of energy, contrast, and correlation were higher than 10% and amounted to 39%, 30,% and 12% respectively, in terms of entropy and homogeneity, they amounted to only 9% and 2%.

## Figures and Tables

**Table 1:** Scheme of composting experiments.

Name of the Experiment ^a^	Volume of the Chamber (dm^3^)	Mass of the Batch Material (kg)
C10N	10	3.6
C10I	10	3.6
C20N	20	7.1
C20I	20	7.1
C50N	50	17.8
C50I	50	17.8
C74N	74	26.3
C74I	74	26.3
C119N	119	42.3
C119I	119	42.3

^a^ Number means the chamber volume in dm^3^, “N” means no additional thermal insulation, “I” means additional insulation.

**Table 2:** Selected material parameters obtained at the end of the composting process.

Name of the Experiment ^a^	Content of Dry Substance (%)	pH	Conductivity (mS)
C10N	16.23	6.7	0.38
C10I	14.56	7.1	0.87
C20N	17.08	6.6	0.38
C20I	16.63	6.1	0.62
C50N	17.73	6.9	0.43
C50I^*^	17.33	8.9	2.07
C74N	15.98	6.6	0.42
C74I^*^	17.95	8.5	1.87
C119N	13.87	6.4	0.40
C119I ^*^	17.40	8.4	1.54

^a^ Number means the chamber volume in dm^3^, “N” means no additional thermal insulation, “I” means additional insulation, ^*^ the composting process was correct.

**Table 3:** Statistical information of the analyzed parameters of color and texture for the images of the material obtained under favorable and unfavorable composting conditions.

Parameter Name	Favorable Conditions		Unfavorable Conditions		p-Value for Mann–Whitney U test
	Average Value	Standard Deviation	Average Value	Standard Deviation	
Color parameters					
R_MEAN	24.86	2.78	42.26	4.30	<0.001
G_MEAN	19.62	2.15	30.39	2.97	<0.001
B_MEAN	15.43	1.81	20.99	2.16	<0.001
R_MEDIAN	19.33	2.12	37.07	4.09	<0.001
G_MEDIAN	14.71	1.51	26.45	2.75	<0.001
B_MEDIAN	11.13	1.18	18.21	2.00	<0.001
GS_MEAN	20.71	2.28	32.86	3.25	<0.001
GS_MEDIAN	15.69	1.58	28.82	3.05	<0.001
WH_PERCENT1	64.69	6.40	92.00	2.96	<0.001
WH_PERCENT2	26.16	5.09	58.77	7.86	<0.001
WH_PERCENT3	12.15	3.64	28.41	6.98	<0.001
WH_PERCENT4	5.17	2.15	12.77	4.43	<0.001
Texture Parameters					
ENERGY	5.50	0.19	6.01	0.19	<0.001
CONTRAST	0.11	0.03	0.14	0.03	<0.001
CORRELATION	0.75	0.03	0.85	0.03	<0.001
ENERGY	0.62	0.08	0.38	0.06	<0.001
HOMOGENEITY	0.95	0.01	0.93	0.01	<0.001
